# Noise-Assisted Instantaneous Coherence Analysis of Brain Connectivity

**DOI:** 10.1155/2012/275073

**Published:** 2012-05-29

**Authors:** Meng Hu, Hualou Liang

**Affiliations:** School of Biomedical Engineering, Science & Health Systems, Drexel University, 3141 Chestnut Street, Philadelphia, PA 19104, USA

## Abstract

Characterizing brain connectivity between neural signals is key to understanding brain function. Current measures such as coherence heavily rely on Fourier or wavelet transform, which inevitably assume the signal stationarity and place severe limits on its time-frequency resolution. Here we addressed these issues by introducing a noise-assisted instantaneous coherence (NAIC) measure based on multivariate mode empirical decomposition (MEMD) coupled with Hilbert transform to achieve high-resolution time frequency representation of neural coherence. In our method, fully data-driven MEMD, together with Hilbert transform, is first employed to provide time-frequency power spectra for neural data. Such power spectra are typically sparse and of high resolution, that is, there usually exist many zero values, which result in numerical problems for directly computing coherence. Hence, we propose to add random noise onto the spectra, making coherence calculation feasible. Furthermore, a statistical randomization procedure is designed to cancel out the effect of the added noise. Computer simulations are first performed to verify the effectiveness of NAIC. Local field potentials collected from visual cortex of macaque monkey while performing a generalized flash suppression task are then used to demonstrate the usefulness of our NAIC method to provide highresolution time-frequency coherence measure for connectivity analysis of neural data.

## 1. Introduction

To understand how brain networks process information, it is crucial to accurately quantify their connectivity patterns. For analysis of brain connectivity between two signals, current measures such as coherence [1–3] rely upon spectral estimate of each signal, which is routinely computed based on Fourier or wavelet transform. Thus, the underlying nonstationary nature of neural data presents a significant challenge for the applications of current measures. Though short-time sliding window approaches, for example, short-time Fourier transform, have been used to alleviate this problem, this issue is not completely resolved for a number of reasons. First, the stationarity of neural data within each short-time window cannot be guaranteed. Second, even though the data are stationary within each time window, the resolution of time-frequency representation is limited by Heisenberg uncertainty principle [4]. Wavelet transform [4], albeit improved, is still subject to time-frequency resolution tradeoff, that is, frequency resolution is low at high frequencies and high at low frequencies. Moreover, wavelet analysis depends on the choice of mother wavelet, which is arbitrary and may not be optimal for time series under scrutiny.

In contrast to the aforementioned spectral estimation methods, empirical mode decomposition (EMD) method [5] adaptively decomposes nonstationary time series into a finite set of amplitude-frequency modulated components, namely, intrinsic mode functions (IMFs), without assuming any basis functions. These IMF components allow the calculation of a meaningful instantaneous frequency by virtue of Hilbert transform. As a result, a high-resolution time-frequency spectral estimation, namely, Hilbert spectrum, can be obtained, even with nonstationary time series. The last decade has witnessed the remarkable success of EMD in a large variety of applications; it is, however, limited to univariate (single-channel) data analysis. The availability of simultaneous multichannel data presents important analysis challenges and calls for multivariate extension of EMD. So far, EMD has been extended to complex EMD [6], rotation-invariant EMD [7], bivariate EMD [8], trivariate EMD [9], multidimensional ensemble EMD [10], and multivariate EMD (MEMD) [11] and its noise-assisted MEMD [12]. Of particular note is the MEMD, which is a rather generic multivariate extension and has been shown very promising in multichannel neural data analysis [13, 14].

Hence, it is natural and straightforward in this study to think of using MEMD together with Hilbert transform to perform spectral estimate of nonstationary multichannel neural data. In practice, the estimated spectra are readily computable, yet its use for subsequent coherence estimate is problematic because MEMD coupled with Hilbert transform provides high-resolution time-frequency spectra typically with many zero values, which therefore cause computational problem for estimating coherence at those zero-value positions.

In this paper, we propose a noise-assisted instantaneous coherence (NAIC) measure based on the MEMD together with the Hilbert transform to circumvent the aforementioned problems in providing high-resolution time-frequency coherence measure. First, the MEMD, together with Hilbert transform, is applied to estimate the spectra of signals. Second, we add a noise into the estimated spectra to alleviate the zero-value problem before coherence is derived. Third, we design a statistical randomization procedure to cancel out the effect of the added noise on the coherence of mixed data. We note that our procedure is not just restricted to the coherence measure demonstrated in this paper, but it can also be applied to other forms of coherence such as partial and multiple coherence as well as Granger causality [15, 16].

The paper is organized as follows. In Section 2, we briefly review the recently developed MEMD method and Hilbert transform, followed by our proposed NAIC method. In Section 3, we first conduct computer simulations to validate our NAIC method and contrast it with both Fourier-based and wavelet-based methods. Then, we apply the method to real cortical filed potential data collected from a macaque monkey while performing a generalized flash suppression task [17].  Section 4 concludes with discussions.

## 2. Method

### 2.1. Background

#### 2.1.1. Multivariate Empirical Mode Decomposition

MEMD is a multivariate extension of EMD. The EMD [5] is a fully adaptive data-driven method which decomposes a time series into a finite set of amplitude-frequency-modulated IMFs, which represent its inherent oscillatory modes. Specifically, for a time series *x*(*t*), all the local extrema are first identified, and then two envelopes *e*_min⁡_(*t*) and *e*_max⁡_(*t*) are obtained by interpolating between local maxima (resp., minima), and subsequently the local mean *m*(*t*) = (*e*_min⁡_(*t*) + *e*_max⁡_(*t*))/2 is computed. The detail *c*(*t*) = *x*(*t*) − *m*(*t*) is finally iterated until it becomes an IMF, which is defined as having the symmetric envelopes and the same numbers of zero-crossing and local extrema, differing at most by one. The residue by removing IMFs from raw signal is subject to the above procedure for the next IMF until the monotonic residue is left. Hence, a time series *x*(*t*) can be expressed as: *x*(*t*) = ∑_*j*=1_^*N*^ *c*_*j*_(*t*) + *r*(*t*), where *c*_*j*_(*t*), *j* = 1,…, *N* are the IMFs, and *r*(*t*) is the residue.

Although the EMD has become an established tool for analysis of single-time series, mode misalignment and mode mixing are two serious problems that limit its further application for multivariate time series. The mode misalignment corresponds to a problem where the same-index IMFs across multivariate data contain different frequency modes so that the IMFs are not matched either in the scale or in the number. The mode mixing occurs when a single IMF contains multiple oscillatory modes and/or a single mode resides in multiple IMFs, which in many cases may obscure the physical meaning of IMFs.

Recently, MEMD has been proposed to alleviate the limitations of EMD and to extend the application of EMD to multivariate time series [11]. An important step in MEMD method is that the calculation of local mean as the concept of local extrema is not well defined for multivariate signals. To deal with this problem, MEMD projects the multivariate signal along different directions to generate multiple multidimensional envelopes; these envelopes are then averaged to obtain the local mean. For an *n*-variable signal, the MEMD algorithm is briefly summarized as follows.

- Construct suitable point set (e.g., the Hammersley sequence) for sampling on an (*n* − 1)- sphere.
- Compute a projection {*p* ^*θ*_*k*_^(*t*)}_*t*=1_^*T*^ of multivariate input data {*v*(*t*)}_*t*=1_^*T*^ along a direction vector *x* ^*θ*_*k*_^ for all *k* giving {*p* ^*θ*_*k*_^(*t*)}_*k*=1_^*K*^.
- Locate the time points *t*_*i*_^*θ*_*k*_^ according to maxima of the set of projected signal {*p* ^*θ*_*k*_^(*t*)}_*k*=1_^*K*^.
- Interpolate [*t*_*i*_^*θ*_*k*_^,*v*(*t*_*i*_^*θ*_*k*_^)] to acquire multivariate envelope curves {*e* ^*θ*_*k*_^(*t*)}_*k*=1_^*K*^.
- Calculate the mean *m*(*t*) of the envelope curves for a set of *K* direction vectors, *m*(*t*) = (1/*K*)∑_*k*=1_^*K*^ *e* ^*θ*_*k*_^(*t*).
- Iterate on the detail *c*(*t*) = *x*(*t*) − *m*(*t*) until it becomes an IMF. The above procedure is applied to the residue *r*(*t*) = *x*(*t*) − *c*(*t*).

The stoppage criterion for multivariate IMF is similar to that for univariate IMFs except that the equality constraint for number of extrema and zero crossings is not imposed, as the extrema cannot be properly defined for multivariate signal.

#### 2.1.2. Hilbert Transform

Hilbert transform [18] has been widely used to obtain analytic (complex) signal associated with a real signal *x*(*t*) and consequently, instantaneous envelope, phase functions and instantaneous frequencies. Given an arbitrary time series *x*(*t*), the corresponding analytic signal is defined as: *z*(*t*) = *x*(*t*) + *iH*[*x*(*t*)] = *a*(*t*)exp⁡[*iθ*(*t*)], where *a*(*t*) and *θ*(*t*) are instantaneous amplitude and phase of the analytic signal *z*(*t*), and the imaginary part *H*[*x*(*t*)] is Hilbert transform of *x*(*t*): *H*[*x*(*t*)] = (1/*π*)*P*[∫_−*∞*_^*∞*^ *x*(*u*)/(*t* − *u*)*du*], where the notation *P* indicates the Cauchy principal value of the integral. The instantaneous frequency can then be obtained from instantaneous phase as: *f*(*t*) = *dθ*(*t*)/*dt*.

Direct application of Hilbert transform to an arbitrary wide-band time series is of little practical value because it could produce negative frequencies, which bear no relationship to real oscillations in a time series [5, 19]. To obtain meaningful and well-behaved instantaneous frequencies, time series to be analyzed must have no riding waves and must be locally symmetrical about its mean as defined by the envelopes of local extrema. According to the definition of IMF, the IMF is an ideal candidate to take full advantage of Hilbert transform. Specifically, given an IMF *c*_*j*_(*t*), we first compute its Hilbert transform *H*[*c*_*j*_(*t*)] and then find its phase through the combination of *c*_*j*_(*t*) and *H*[*c*_*j*_(*t*)]. The instantaneous frequency of IMF is finally obtained as the derivative of the instantaneous phase with respect to time. As such, we can apply Hilbert transform to the decomposed IMFs from a time series and construct a time-frequency analytic (complex) matrix, whose absolute value is the well-known Hilbert spectrum [5]. The resulting time-frequency analytic matrix makes it possible to calculate cross-spectrum between signals and autospectra of individual signals, which form the basis for coherence estimation.

### 2.2. Noise-Assisted Instantaneous Coherence

**Figure 1:**
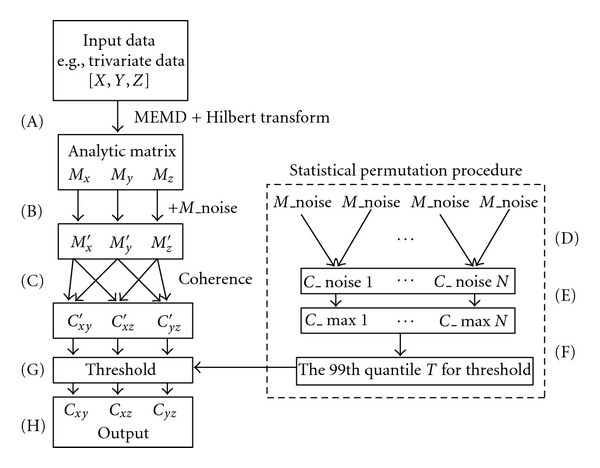
Schematic representation of the proposed noise-assisted instantaneous coherence (NAIC). A trivariate data [X Y Z] is used as an example. The first step (A) consists of transforming each time series to the corresponding analytic matrix by virtue of the MEMD and Hilbert transform. A random noise complex matrix is then added to the analytic matrix of data (B) to facilitate the calculation of coherence (C). Two random noise complex matrices are independently generated to compute their coherence. The process is repeated for *N* (e.g., 1000) times (D) to obtain a null distribution of the maximum coherence (E). Here, we set the *P* value as 0.01, thus the threshold “*T*” corresponds to the 10th value from the maximum of the null distribution (F). Finally, we use the “*T*” to threshold the coherence from (C) to be considered as statistically significant from noise (G). The output of NAIC (H) provides high-resolution time-frequency coherence spectrum.

Conventional coherence methods based on Fourier transform or autoregressive model assume that input signals are stationary, and their time-frequency representations suffer from the fundamental uncertainty principle. In this study, we propose a noise-assisted instantaneous coherence, which is suited to the analysis of nonstationary neural signals and offers high-resolution time-frequency coherence estimate. A schematic representation of the processing steps is shown in Figure 1.

In this method, we first employ the MEMD to adaptively decompose raw neural data into IMFs. Before applying MEMD, it should be noted that (1) neural data are often collected over certain time period from multiple channels across many trials, which can be represented as a three-dimensional matrix, that is, TimePoints × Channels × Trials, on which the MEMD cannot be directly applied, and (2) neural recordings are usually of high degree of variability, typically collected over many trials spanning from days to months, or even years, which has significant detrimental impact upon the final decomposition of MEMD when projecting the data in multidimensional space. Therefore, two important preprocessing steps [14] should be taken before applying the MEMD to neural data. First, high-dimensional neural data (e.g., TimePoints × Channels × Trials) is reshaped into such a two-dimensional time series as TimePoints × [Channels × Trials] before submitted for the MEMD analysis. It is an important step to make sure that all the IMFs be aligned not only across channels but also across trials. Second, in order to reduce the variability among neural recordings, individual time series is normalized against its temporal standard deviation before the MEMD is applied and subsequently restores the standard deviations to the corresponding IMFs after the MEMD.

Once Hilbert transform is applied to the obtained IMFs, each signal of interest yields a time-frequency analytic (complex) matrix. As described above, the analytic matrix typically exhibits many zero values due to its high resolution, which thus cause the computational issue. As such, before computing coherence, we add a random noise complex matrix to the analytic matrix of raw data to eliminate the zero values. The real and imaginary parts of the added noise complex matrix are set to normally distributed random noise. Coherence is then estimated based on the mixed time-frequency analytic matrix. The coherence between signals *i* and *j* is defined as [20]: $C_{ij}\left(f\right)=\left|S_{ij}\left(f\right)\right|/\sqrt{S_{ii}\left(f\right)S_{jj}\left(f\right)}$, where *S*_*ii*_(*f*) and *S*_*jj*_(*f*) are the autospectra of signals, and *S*_*ij*_(*f*) is the cross-spectrum between signals. In this noise-assisted procedure, the added noise should be of small magnitude so as to minimize the interaction between the added noise and the original clean signal. To account for the effect of the added noise on the estimated coherence, a statistical randomization procedure is designed as follows.

1. Time-frequency coherence of two random noise complex matrices is estimated.
2. The maximum value of coherence across all the time and frequency in (1) is collected.
3. Repeat Steps (1)-(2) many times, for example, 1000 times, to obtain a null distribution of the maximum coherence.
4. From the experimental value of the estimated coherence and the aforementioned null distribution, calculate the proportion of maximum coherence in the null distribution that is larger than the experimentally observed. This proportion is called the *P* value.
5. The experimental value of the estimated coherence is considered to be significant if the *P* value is smaller than the critical alpha level, for example, 0.05.

This procedure is a nonparametric randomization test [21], which does not need to perform statistical test at each time-frequency location, thus bypasses multiple comparison problems. By having followed all these steps, the NAIC can provide a high-resolution time-frequency coherence representation. Note that our proposed NAIC method can be readily extended to other forms of coherence such as partial and multiple coherence [20, 22] as well as Granger causality measure [16].

## 3. Results

### 3.1. Simulations

**Figure 2:**
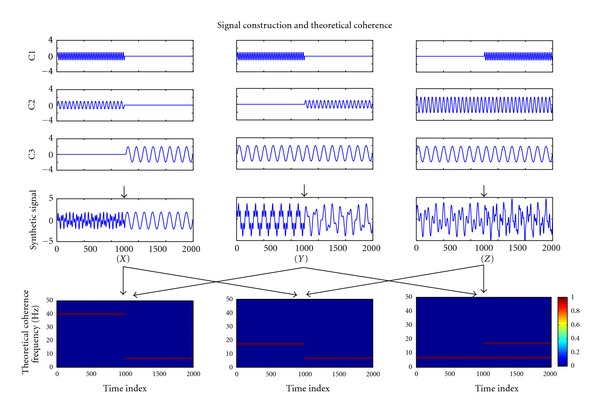
Construction of the synthetic trivariate nonstationary signal [X Y Z] and their theoretic coherence between channels. In this figure, the first three rows (C1, C2, and C3) show the components used to generate the synthetic data (the fourth row). The last row shows theoretical coherence between different channels of the synthetic data.

In this simulation, we generated a nonstationary three-channel signal [X, Y, Z] by concatenating and superposing three sinusoid waves, each with different frequency.  Figure 2 showed how nonstationary three-channel signal was constructed and its theoretical coherence between channels. We used this synthetic signal to verify the effectiveness of our NAIC in offering a high-resolution time-frequency coherence spectrum of nonstationary time series.

**Figure 3:**
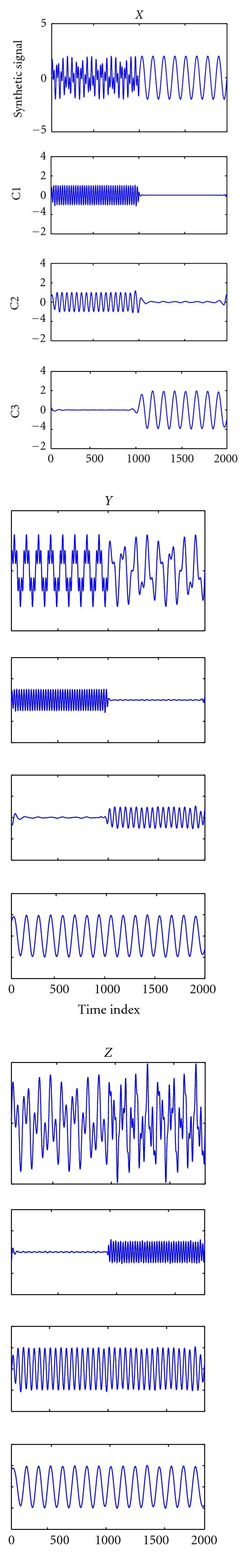
Decomposition of a synthetic trivariate nonstationary signal [X Y Z] via MEMD. The decomposed three IMFs C1–C3 correctly recover the designed components in the data (Figure 2).

**Figure 4:**
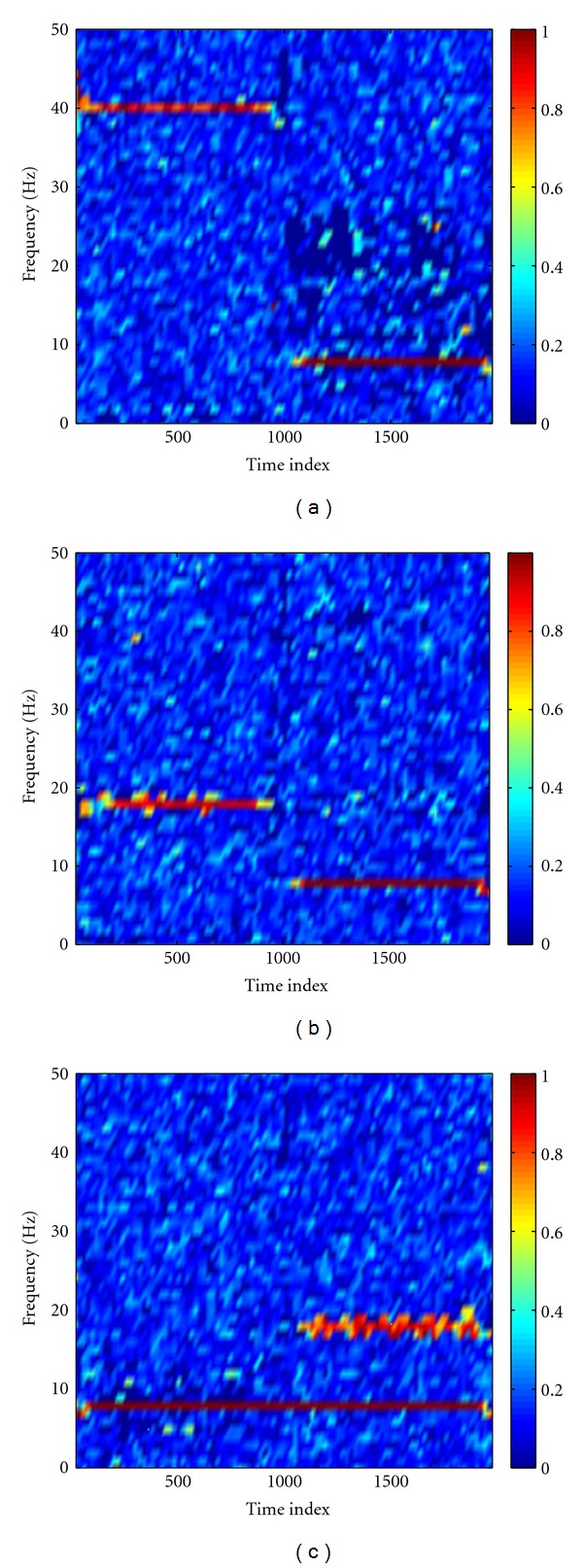
Time-frequency coherence based on the mixed analytic matrix for X-Y (a), X-Z (b), and Y-Z (c). A random noise complex matrix is superposed to the analytic matrix of data for facilitating the calculation of coherence. From these plots, we can see that the obtained coherence reflects the designed coupling patterns in the synthetic signals but contains a lot of artifacts scattered in the spectra.

**Figure 5:**
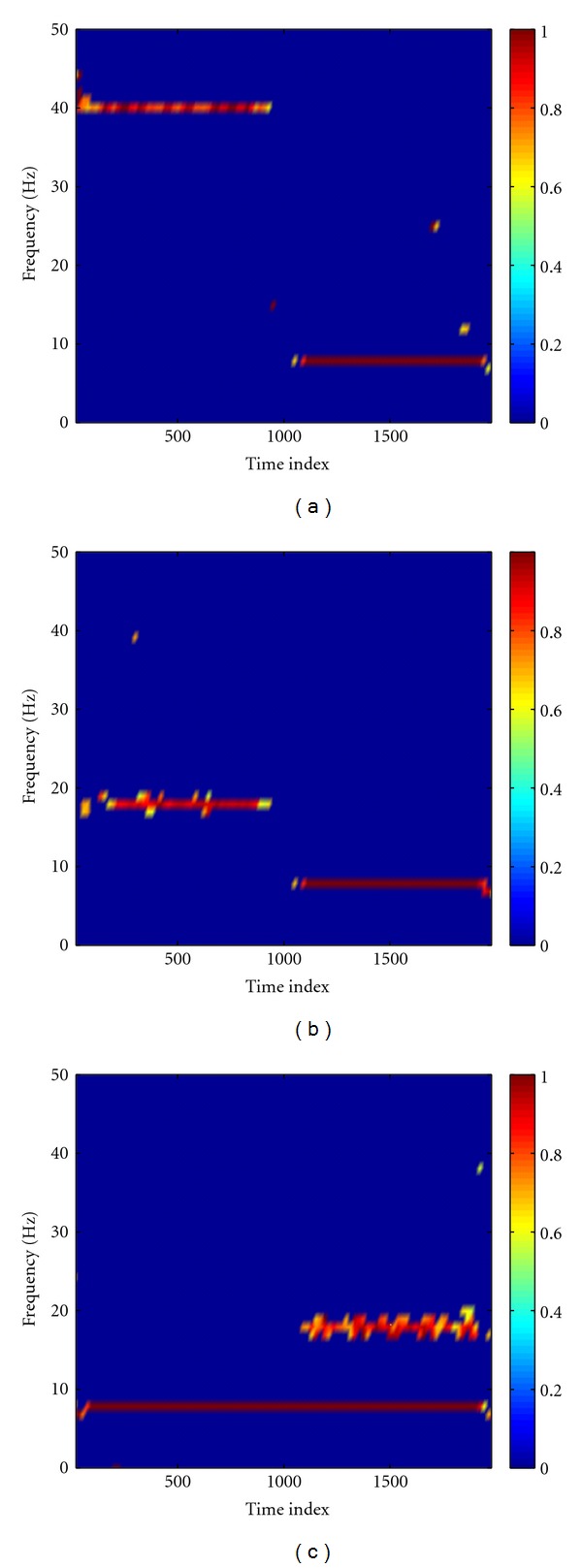
The noise-assisted instantaneous coherence (NAIC) for X-Y (a), X-Z (b), and Y-Z (c). From these plots, we can see that the designed coupling patterns in the simulation (Figure 2) are well captured by our NAIC method.

**Figure 6:**
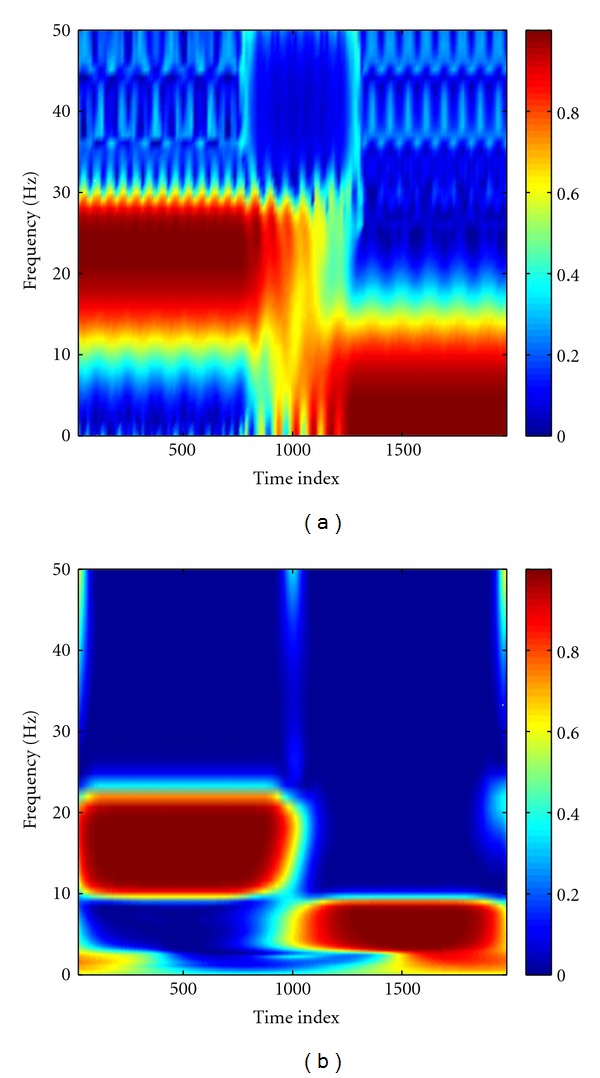
Fourier- (a) and wavelet- (b) based coherence for X-Z in the simulation. The coherence between X and Z is used as an example to demonstrate that our NAIC method (Figure 5) can provide better time-frequency resolution than Fourier- and wavelet-based coherence estimations.

 The MEMD was first performed to decompose the synthetic nonstationary data.  Figure 3 showed that the raw data were decomposed into three IMFs, which correctly recovered the designed components in the data (Figure 2), and the common modes within the data were aligned in the IMFs with the same index. By virtue of Hilbert transform, each time series was represented by a time-frequency analytic matrix. A random noise complex matrix with noise variance of 10^−4^ was then superposed to the analytic matrix of clean data so as to facilitate the calculation of coherence between channels.  Figure 4 showed time-frequency coherence based on the mixed data. From this figure, we can see that the obtained time-frequency coherence spectra reflect the designed coupling between channels. We notice, however, that the added noise induces some artifacts, shown as the bright spots scattered in the spectra. We subsequently performed the statistical randomization procedure in which the noise variance is set to 10^−4^ to identify statistically significant coherence. In Figure 5, we showed that significant coherence (*P* < 0.01) in the simulation (Figure 2) was well captured by our NAIC method. As comparisons, Fourier- and wavelet-based coherence methods were, respectively, performed to analyze the same synthetic data. For wavelet-based coherence, we used the “Morlet” as the mother wavelet (other wavelets yield very similar results). As an example, time-frequency coherence spectra based on the Fourier and wavelet transform between channel X and Z were shown in Figure 6, in which we can see that both coherence spectra exhibit poor time-frequency resolution relative to the proposed NAIC.

**Figure 7:**
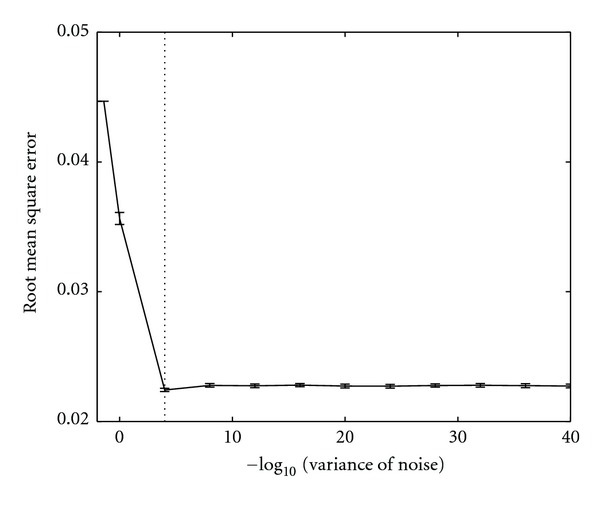
Root mean square error (RMSE) between the estimated coherence and its theoretical value as a function of the noise variance. Error bars denote standard deviations over 50 repetitions. Note that the RMSE declined as the noise variance decreased and stayed constant once the noise variance approaches 10^−4^.

In our NAIC approach, an important question is how much noise is acceptable. To examine the effect of noise on coherence estimation, we systematically varied the noise by changing its variance relative to the signal and estimated the coherence between channel X and Z in the above simulation. We measured the root mean square error (RMSE) [23] between the estimated coherence and its theoretical value as a function of noise variance. We repeated the same analysis procedure for 50 times to obtain error bars at each noise level. The result is shown in Figure 7. We can see from the figure that the RMSE declined as the noise variance decreased, and stayed constant when the noise variance approaches 10^−4^. While the amount of noise derived from this particular simulation is empirical, it indicates that the amount of noise should be four orders of magnitude less than the signal. As a rule of thumb, we suggest that the added noise should be of infinitesimal magnitude so as to minimize the interaction between the added noise and original clean signal.

### 3.2. Noise-Assisted Instantaneous Coherence Analysis of Cortical Field Potential Data

 In this section, we used local field potentials (LFPs) collected from visual cortex of macaque monkey while performing a visual illusion task as an example to demonstrate the usefulness of our proposed NAIC approach in providing high-resolution time-frequency coherence spectrum for nonstationary neural data.

 The visual illusion task used here is called generalized flash suppression (GFS), in which a salient visual stimulus could be rendered invisible despite continuous retinal input. It provides a rare opportunity to study neural mechanisms directly related to perception [17]. In the GFS task, after the monkey gained fixation, target stimulus was presented for 1400 msec and immediately followed by the surroundings stimuli. With the presence of the surroundings, the target could be rendered subjective invisible. The monkey was trained to respond to the visibility conditions such that the trial was classified as either “Visible” or “Invisible”. Note that the stimuli in these two conditions were physically identical. Multielectrode LFP recordings were simultaneously collected from multiple cortical areas V1, V2, and V4 while monkeys performed the GFS task. The data were obtained by band pass filtering the full bandwidth signal between 1 and 500 Hz and then resampled at 1 KHz [24]. In this study, two-channel LFP from area V1 of one-second long after surrounding onset over 65 trials was used for demonstration.

**Figure 8:**
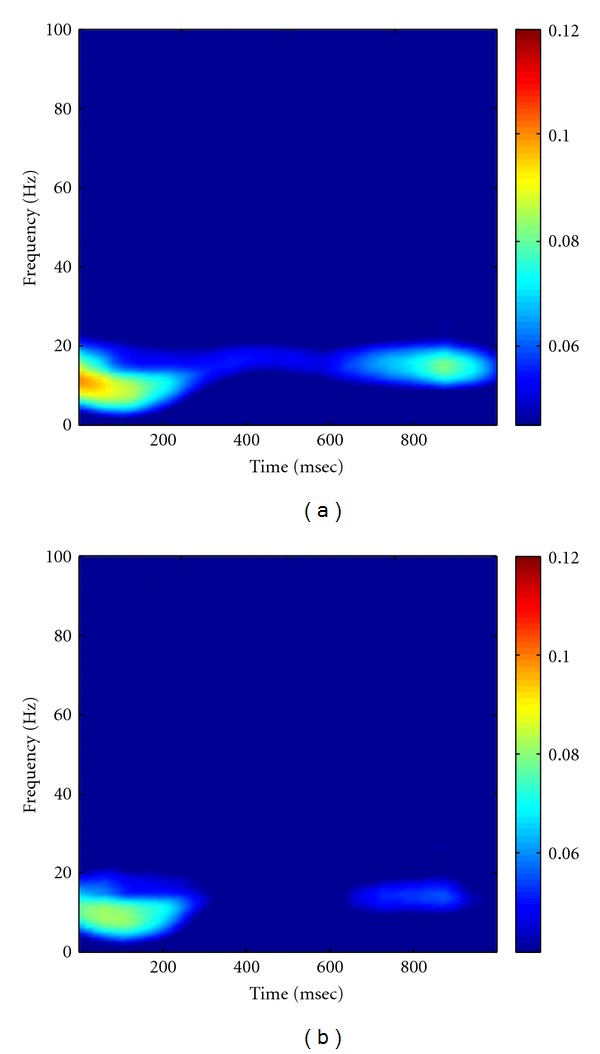
Grand average of the smoothed NAIC spectra in Visible (a) and Invisible (b) conditions. In comparison of (a) with (b), we can see that Visible condition exhibits larger coherence than Invisible condition at round 400 msec after surrounding onset.

**Figure 9:**
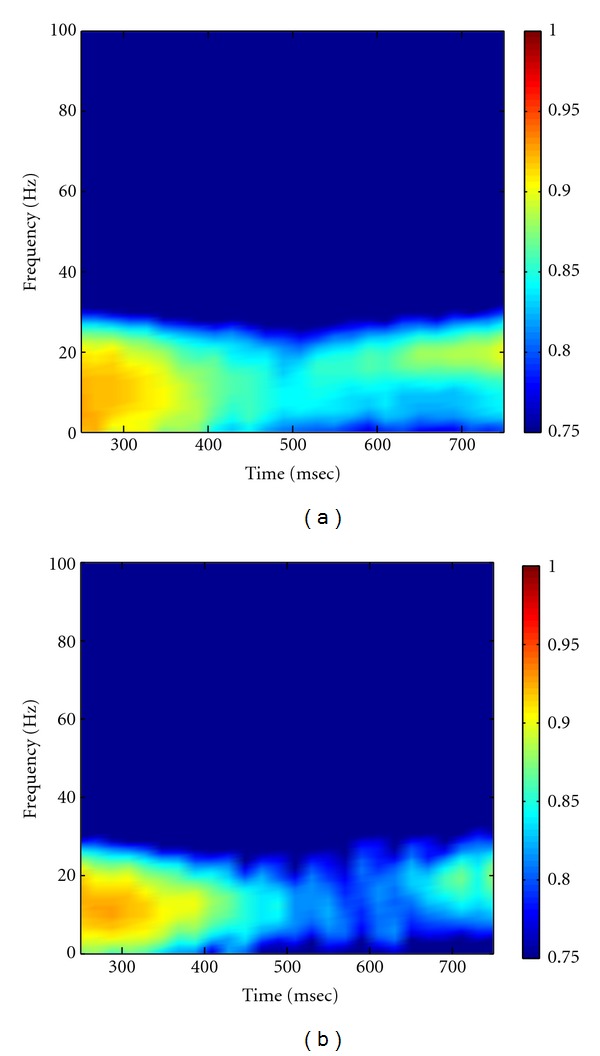
Grand average of Fourier-transform-based coherence in Visible (a) and Invisible (b) conditions. Note that the time-evolving coherence was obtained using a moving window approach, in which the window size used was 500 msec long, with a step size of 10 msec. As a result, coherence was only displayed between 250–750 msec.

**Figure 10:**
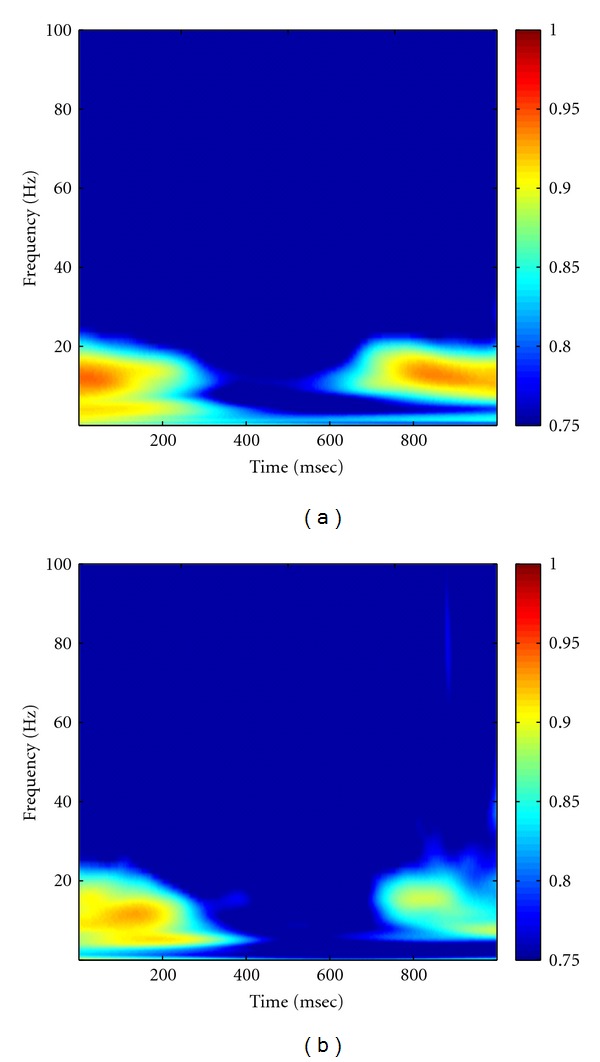
Grand average of wavelet-transform-based coherence in Visible (a) and Invisible (b) conditions. Note that we used the “Morlet” as the mother wavelet.

**Figure 11:**
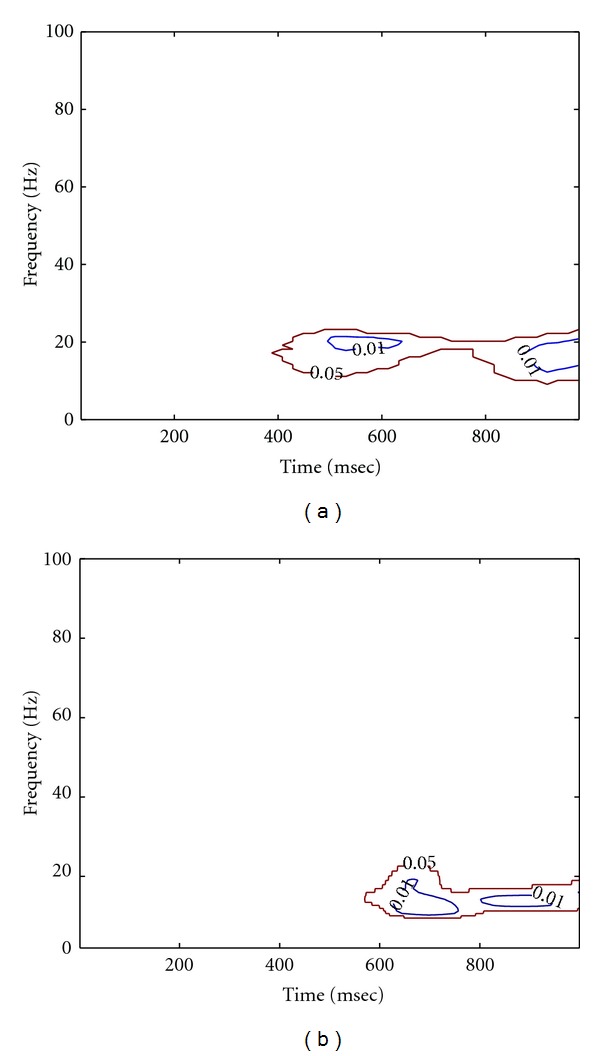
Significance test of difference between two perceptual conditions revealed by the NAIC (a) and the wavelet-based method (b). General agreement of two methods is evident, yet the NAIC is able to detect statistically significant difference of perceptual suppression occurring as early as 400 msec after surrounding onset. Level lines are depicted at *P* < 0.05 (red) and *P* < 0.01 (blue), respectively.

As described in Method part, the MEMD was first performed on multichannel multitrial LFP data to produce the IMFs, followed by Hilbert transform to obtain the analytic matrix of data. A random noise matrix with noise variance of 10^−4^ was then added to the analytic matrix of data to facilitate the calculation of coherence. The high-resolution time-frequency coherence spectrum was finally obtained by applying the proposed statistical randomization procedure in which the noise variance was set to 10^−4^. Figures 8(a) and 8(b) showed the grand average of the NAIC spectra in the Visible and Invisible conditions, respectively. From this figure, we can see clearly that the 10 Hz coherence initially appeared in both conditions for about 200 msec after the surrounding onset. We then observed a slightly shift of oscillatory frequency to 10–20 Hz with reduced coherence, yet the Visible condition exhibited greater coherence than the Invisible condition. As comparisons, we applied Fourier- and wavelet-based coherence methods to the same neural data, with results shown in Figures 9 and 10, respectively. Based on these figures, we can see that Fourier- and wavelet-based methods exhibited similar coherence patterns but with poor time-frequency resolution. Furthermore, we compared the NAIC spectra between Invisible and Visible conditions to reveal how neural connectivity reflected perceptual suppression. We initially performed point-wise significance test by applying *t*-test to every time-frequency index between two conditions. As shown in Figure 11(a), significant perceptual suppression effect was evident in about 400 msec after the surrounding onset between 10 and 20 Hz, in which Visible condition showed significantly larger coherence than Invisible condition (*P* < 0.05, uncorrected). To deal with multiple-comparison problem, for which several methods have been proposed [21, 25, 26], we adopted a clustered-based nonparametric method [21] and found that the significant difference observed between two conditions still survived (*P* < 0.05). For comparison, we repeated the same statistical procedure to the wavelet-based coherence between two conditions. The resulting significant difference at both *P* < 0.05 and *P* < 0.01 is shown in Figure 11(b). Both NAIC and wavelet methods show general agreement about the concentration of significant difference in frequency. However, the NAIC is more sensitive in revealing significant difference of perceptual suppression that occurred as early as 400 msec after surrounding onset. These results together suggest that neural coherence reflects perceptual suppression, and significantly reduced coherence in Invisible condition may be associated with the reduction of brain connectivity.

## 4. Discussion

In this paper, we introduced a noise-assisted instantaneous coherence (NAIC) to achieve high-resolution time-frequency coherence measure. In our method, the fully data-driven MEMD, together with Hilbert transform, was first employed to provide high-resolution time-frequency spectral representation for nonstationary neural data. We then added random noise onto the spectra, which makes the calculation of coherence measure feasible. Finally, a statistical randomization procedure was designed to identify the statistically significant coherence. Computer simulations confirm that our NAIC is effective for coherence analysis of nonstationary signal. Cortical LFP data further demonstrates that our NAIC method indeed is able to provide a high-resolution time-frequency coherence representation for connectivity analysis of neural data.

The use of noise in data analysis has long been known. There are only a few that are relevant to EMD analysis. Broadly, there are two ways to utilize noise for EMD analysis. One is to assign statistical significance of information content for IMF components from any noisy data by exploiting numerical observations that (1) EMD of white noise acts essentially as a dyadic filter [27], and (2) all the IMFs of white noise follow a normal distribution [28]. Another way is to improve the EMD method by adding noise to the data. Early attempt has been made to add noise of infinitesimal amplitude to the data short of extrema in order to make the EMD operable [29]. Wu and Huang [30] explored the benefit of dyadic filter bank structure of EMD for white noise and proposed ensemble EMD (EEMD) in which multiple realizations of white noise are added to the data before applying EMD. The effect of the added white noise is to provide a uniformly distributed reference scale, which enables EMD to preserve the dyadic property and hence reduce the chance of mode mixing. Given the random effect of noise in multiple realizations, added noise is eventually canceled out in the ensemble mean. Recently proposed noise-assisted MEMD (NA-MEMD) [12], similar to EEMD, also makes use of the dyadic property to reduce the mode-mixing problem; however, unlike EEMD, it adds white noise as separate channels and thus only a single sweep of MEMD is applied.

Our NAIC method is radically different from the above methods in that the noise is introduced *after* MEMD data decomposition. In the procedure, the noise is added to the Hilbert spectrum of data derived from MEMD to eliminate the zero values in the spectral representation and thus make the coherence estimation operable. The effect of the added noise is eliminated via a statistical randomization procedure. How much noise should be added is a crucial question in any noise-assisted methods. In our method, as a rule of thumb, we suggest that the added noise should be of infinitesimal magnitude so as to minimize the interaction between the added noise and original clean signal.

We note that our procedure is not just limited to the coherence measure demonstrated in this paper, but it can also be used for other forms of coherence estimation such as partial and multiple coherence as well as Granger causality [15, 16]. In addition, it is conceivable that our procedure could be applied to phase-based measures including phase synchrony [31] and even phase-based causality measure [32, 33]. Phase synchrony based on EMD [31] has clear advantage in adaptively extracting the narrow-band components (IMF) of signal and thus avoiding arbitrary preselection of frequency ranges. Importantly, phase synchrony could be used to reveal potential nonlinear coupling between IMFs of different scales, which makes the approach very attractive. We should note that Hilbert transform is usually used to estimate instantaneous phase, which can also be calculated by alternative methods [34].

A significant strength of our coherence estimation is that the data stationarity is not required. Despite its promise, coherence is essentially a linear measure, which may fail to capture underlying nonlinear relations. As such, phase synchrony may offer a viable solution to circumvent this issue. A detailed comparison of our method with phase synchrony against well-characterized neural data would serve to identify their relative strengths and weaknesses. In addition, when signal-to-noise ratio is low, special care should be taken to interpret the estimation of coherence which could become unreliable. Furthermore, while analyzing large amount of neural data, there is a particular concern about how to reduce the computational load. Nonetheless, we have presented in the paper a noise-assisted data analysis method to achieve high-resolution coherence estimation. The analysis is supported by simulations on both synthetic and real neural data.
